# Validation of Fetal Medicine Foundation charts for fetal growth in twins: nationwide Danish cohort study

**DOI:** 10.1002/uog.29125

**Published:** 2024-10-27

**Authors:** S. E. Kristensen, A. Wright, D. Wright, K. Gadsbøll, C. K. Ekelund, P. Sandager, F. S. Jørgensen, E. Hoseth, L. Sperling, H. J. Zingenberg, K. Sundberg, A. McLennan, K. H. Nicolaides, O. B. Petersen

**Affiliations:** ^1^ Center for Fetal Medicine, Pregnancy and Ultrasound, Department of Gynecology, Fertility and Obstetrics Copenhagen University Hospital Rigshospitalet, Copenhagen Denmark; ^2^ Faculty of Health and Medical Sciences, Department of Clinical Medicine University of Copenhagen Copenhagen Denmark; ^3^ Faculty of Health and Medical Sciences, Institute of Cellular and Molecular Medicine University of Copenhagen Copenhagen Denmark; ^4^ Institute of Health Research University of Exeter Exeter UK; ^5^ Department of Obstetrics and Gynecology, Center for Fetal Medicine Aarhus University Hospital Aarhus Denmark; ^6^ Center for Fetal Diagnostics Aarhus University Hospital Aarhus Denmark; ^7^ Department of Clinical Medicine Aarhus University Aarhus Denmark; ^8^ Department of Obstetrics and Gynecology, Fetal Medicine Unit Copenhagen University Hospital – Hvidovre and Amager Hvidovre Denmark; ^9^ Department of Obstetrics and Gynecology, Clinic of Ultrasound Aalborg University Hospital Aalborg Denmark; ^10^ Department of Obstetrics and Gynecology, Center for Ultrasound and Pregnancy Odense University Hospital Odense Denmark; ^11^ Department of Obstetrics and Gynecology, Ultrasound in Pregnancy Copenhagen University Hospital – Herlev and Gentofte Herlev Denmark; ^12^ Sydney Ultrasound for Women Chatswood NSW Australia; ^13^ Discipline of Obstetrics, Gynaecology and Neonatology The University of Sydney Sydney NSW Australia; ^14^ Harris Birthright Research Centre for Fetal Medicine King's College Hospital London UK

**Keywords:** dichorionic, external validation, fetal growth model, growth monitoring, monochorionic diamniotic, national cohort, singleton reference distribution, small‐for‐gestational age, twin pregnancy, twin reference distribution

## Abstract

**Objective:**

To assess the validity of the Fetal Medicine Foundation (FMF) chorionicity‐specific models for fetal growth in twin pregnancy.

**Methods:**

This was an external validation study of the FMF models using a nationwide Danish cohort of twin pregnancies. The cohort included all dichorionic (DC) and monochorionic diamniotic (MCDA) twin pregnancies with an estimated delivery date between 2008 and 2018, which satisfied the following inclusion criteria: two live fetuses at the first‐trimester ultrasound scan (11–14 weeks' gestation); biometric measurements available for the calculation of estimated fetal weight (EFW) using the Hadlock‐3 formula; and delivery of two liveborn infants. Validation involved assessing the distributional properties of the models and estimating the mean EFW *Z*‐score deviations. Additionally, the models were applied to pregnancies that delivered preterm and attended non‐scheduled visits (complicated pregnancies).

**Results:**

Overall, 8542 DC and 1675 MCDA twin pregnancies met the inclusion criteria. In DC twins, 17 084 fetuses were evaluated at a total of 95 346 ultrasound scans, of which 44.5% were performed at scheduled visits in pregnancies carried to 37 + 0 weeks or later. The median number of growth scans per DC twin fetus from 20 + 0 weeks onwards was four. The model showed good agreement with the validation cohort for scheduled visits in DC twins delivered at 37 + 0 weeks or later (mean ± SD EFW *Z*‐score, –0.14 ± 1.05). In MCDA twins, 3350 fetuses underwent 31 632 eligible ultrasound scans, of which 59.5% were performed at scheduled visits in pregnancies carried to 36 + 0 weeks or later. The median number of growth scans per MCDA twin fetus from 16 + 0 weeks onwards was 10. The model showed favorable agreement with the validation cohort for scheduled visits in MCDA twins delivered at 36 + 0 weeks or later (mean ± SD EFW *Z*‐score, –0.09 ± 1.01). Non‐scheduled visits and preterm delivery before 37 + 0 weeks for DC twins and before 36 + 0 weeks for MCDA twins corresponded with smaller weight estimates, which was consistent with the study's definition of complicated pregnancy.

**Conclusions:**

The FMF models provide a good fit for EFW measurements in our Danish national cohort of uncomplicated twin pregnancies assessed at routine scans. Therefore, the FMF models establish robust criteria for subsequent investigations and potential clinical applications. Future research should focus on exploring the consequences of clinical implementation, particularly regarding the identification of twins that are small‐for‐gestational age, as they are especially susceptible to adverse perinatal outcome. © 2024 The Author(s). *Ultrasound in Obstetrics & Gynecology* published by John Wiley & Sons Ltd on behalf of International Society of Ultrasound in Obstetrics and Gynecology.

**CONTRIBUTION**

*What are the novel findings of this work?*

This study used a nationwide Danish cohort of 8542 dichorionic and 1675 monochorionic diamniotic twin pregnancies to externally validate the Fetal Medicine Foundation (FMF) chorionicity‐specific models for fetal growth in twin pregnancies compared with singleton percentiles. The dual‐percentile scale may offer improved resolution compared with the conventional scale in g.

*What are the clinical implications of this work?*

The FMF chorionicity‐specific models provide a way of assessing fetal growth in twins relative to singletons. Future research should seek to improve the accuracy, validity and clinical utility of these models for growth monitoring in twin pregnancy.

## INTRODUCTION

Twin pregnancies present unique challenges in obstetrics, necessitating tailored monitoring and antenatal care to ensure optimal outcomes for the mother and the twins. Accurate assessment of fetal growth is crucial in managing twin pregnancy, but it remains complex owing to inherent physiological variations and pathological conditions associated with multifetal pregnancy and chorionicity. Twin pregnancies account for 1.4% of all pregnancies in Europe^1^. However, the proportion of twins requiring admission to the neonatal intensive care unit is much higher than that for singletons, partly owing to the higher proportion of infants being born preterm but also because of impaired fetal growth and the risks associated with low birth weight^2^, ^3^, ^4^, ^5^.

In conventional obstetric practice, the categorization of fetal development into small‐for‐gestational age (SGA), appropriate‐for‐gestational age (AGA) or large‐for‐gestational age was based on standard growth charts developed for singleton pregnancies. These growth charts serve as universal references, based on the assumption that a fetus has the same genetic growth potential regardless of the plurality of the pregnancy, thereby facilitating a uniform methodology for assessing fetal growth across all pregnancies and enabling comparisons with the general population^6^, ^7^, ^8^, ^9^.

**Figure 1:**
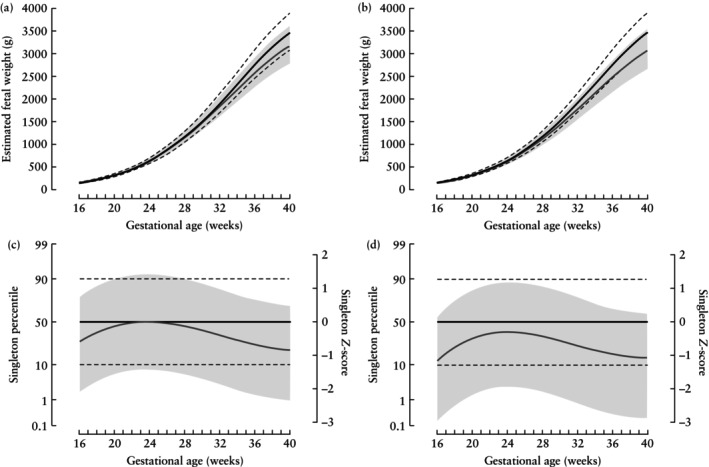
Fetal Medicine Foundation chorionicity‐specific fetal growth charts for dichorionic (a,c) and monochorionic diamniotic (b,d) twins, in relation to singleton estimated fetal weights, percentiles and *Z*‐scores, reproduced from Wright *et al*.^10^. Solid black line is 50^th^ percentile and black dashed lines are 10^th^ and 90^th^ percentiles for singletons. Solid gray line is 50^th^ percentile and shaded area represents range between 10^th^ and 90^th^ percentiles for twins.

The Fetal Medicine Foundation (FMF) has recently introduced a reference for twins on a singleton percentile scale^10^. This enables growth in twins to be assessed against both singleton and twin reference distributions (Figure 1). The additional information on fetal growth provided by this reference compared with a twin‐specific reference could offer a better understanding of the complexities associated with these high‐risk pregnancies^11^, ^12^, ^13^, ^14^.

The objective of this study was to externally validate the FMF models for describing chorionicity‐specific fetal growth in twin pregnancies relative to that in singleton pregnancies.

## METHODS

### Study population and design

This was an external validation study of the FMF models using the nationwide Danish cohort of twin pregnancies. The cohort included all dichorionic (DC) and monochorionic diamniotic (MCDA) twins with an estimated delivery date between 1 January 2008 and 31 December 2018 that satisfied the inclusion criteria listed below. The cohort was based on national highly detailed data from all local obstetric departments stored in the five regional Astraia servers^15^ combined with data obtained from the Danish Fetal Medicine Database over a span of 11 years, as described previously^16^, ^17^. In Denmark, the public healthcare system provides ultrasound scans and first‐trimester aneuploidy screening to all pregnant women between 11 and 14 weeks' gestation. The participation rate for this screening program exceeded 95% of all pregnant women during the study period^18^. Determination of chorionicity is performed for every twin pregnancy in the first trimester by evaluating the placentae and the intertwin membrane for the presence of the lambda sign or the T‐sign^19^, ^20^. Additionally, obstetric departments across Denmark consistently comply with national guidelines for the management of twin pregnancies. These guidelines include a recommendation for scheduled visits at 4‐weekly intervals for uncomplicated DC twins starting from 20 weeks, and at 2‐weekly intervals for uncomplicated MCDA twins commencing from 16 weeks. Furthermore, the guidelines recommend delivery of DC twins at approximately 37–38 weeks and of MCDA twins at around 36–37 weeks.

Inclusion criteria for the study were a pregnancy with two live fetuses at the first‐trimester ultrasound scan resulting in the birth of two liveborn infants. Exclusion criteria included terminated pregnancy, miscarriage before 24 + 0 weeks, stillbirth at or after 24 + 0 weeks, intrauterine demise of one fetus, pregnancy undergoing fetal reduction, missing outcome information or inadequate growth assessment to calculate estimated fetal weight (EFW) using the Hadlock‐3 formula^21^, ^22^. Pregnancies affected by twin‐specific complications such as selective fetal growth restriction, twin–twin transfusion syndrome or twin anemia polycythemia sequence were not specifically excluded, unless they did not fulfill the inclusion criteria. Cases in which chorionicity was uncertain and could not be determined through electronic medical records were excluded from further analysis. These exclusion criteria were chosen to align with those used in the development cohort^10^. Abnormal Doppler measurements were not included in the development cohort, thus they were not included in this study.

The study received approval from the regional authority responsible for data security management (approval number: P‐2020‐381) and the Danish clinical quality registry (RKKP‐case number: FØTO‐2021‐03‐22). The Danish Patient Safety Authority authorized the retrieval of missing or supplementary data (case number: 31‐1521‐356). It should be noted that, following Danish legislation, ethical approval is not required for registry‐based studies.

### Outcome measures

The study defined uncomplicated DC and MCDA twin pregnancies as those seen for scheduled visits, resulting in two liveborn children delivered at or after 37 + 0 weeks in DC twin pregnancies and at or after 36 + 0 weeks in MCDA twin pregnancies. These cut‐offs were determined based on international guidelines for the recommended timing of delivery in twin pregnancies^23^, ^24^. Scheduled visits were defined as those occurring at 4‐weekly intervals in DC twins from 20 weeks' gestation and at 2‐weekly intervals in MCDA twins from 16 weeks^23^, ^24^. Non‐scheduled visits refer to those that took place between the scheduled visits. Pregnancies with non‐scheduled visits and those that delivered preterm were considered as complicated.

EFW was calculated using the Hadlock‐3 formula, using head circumference, abdominal circumference and femur length^21^, ^22^. EFW values were normalized as *Z*‐scores (adjusted by gestational age) and calculated using the FMF reference for singletons^6^.

### Statistical analysis

Chorionicity‐specific fetal growth in twins described by the FMF models^10^, relative to singletons, is illustrated in Figure 1. The models were developed by fitting Bayesian hierarchical models using Markov chain Monte Carlo (MCMC) simulation methods^25^. Moreover, the models employed a hierarchical Gaussian framework with three levels: (1) pregnancy; (2) fetus; and (3) visit. The models were fitted to singleton *Z*‐scores for uncomplicated DC and MCDA twin pregnancies.

For the primary validation of the models, a subgroup of uncomplicated twin pregnancies in the validation cohort was used. For comparison, an assessment was also made of non‐scheduled visits and pregnancies that delivered before 37 + 0 weeks in DC twins or before 36 + 0 weeks in MCDA twins. This assessment aimed to assess deviations from the FMF reference in non‐scheduled growth assessments and in pregnancies that delivered preterm. Summary statistics and Gaussian probability plots of *Z*‐scores for EFW in MCDA and DC twins were generated to assess the accuracy of the models. The *Z*‐scores were distributed from the models for scheduled and non‐scheduled visits according to term or preterm delivery and for each scheduled visit in both chorionicities. Data analysis was carried out using the statistical software RStudio (version 2023.06.0+421)^26^.

## RESULTS

**Figure 2:**
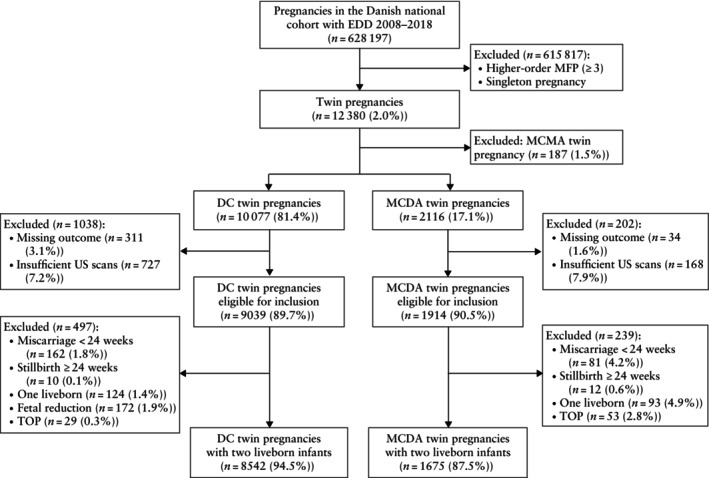
Flowchart summarizing inclusion of patients in study population. DC, dichorionic; EDD, estimated delivery date; MCDA, monochorionic diamniotic; MCMA, monochorionic monoamniotic; MFP, multifetal pregnancy; TOP, termination of pregnancy; US, ultrasound.

In the cohort of 12 380 twin pregnancies, 10 077 (81.4%) were DC, of which 8542 (84.8%) met the inclusion criteria, and 2116 (17.1%) were MCDA, of which 1675 (79.2%) met the inclusion criteria^27^. Reasons for exclusion are given in Figure 2.

### Baseline characteristics

Maternal baseline characteristics and obstetric history are summarized in Table 1. Most women were in their late twenties or early thirties. There were few active smokers and approximately half of the women were nulliparous. The majority identified as Caucasian and had a pregestational body mass index within the normal range. Natural conception accounted for half of the DC pregnancies compared with 85.0% of the MCDA pregnancies.

**Table 1:** Baseline maternal characteristics of twin pregnancies included in validation cohort

Characteristic	DC twin pregnancy (*n* = 8542)	MCDA twin pregnancy (*n* = 1675)
Fetuses (*n*)	17 084	3350
Maternal age (years)	32 (29–35)	30 (27–33)
Maternal height (cm)*	169 (164–173)	168 (164–172)
Maternal weight (kg)*	67 (60–78)	65 (59–76)
Prepregnancy BMI (kg/m^2^)*	24 (21–27)	23 (21–27)
Conception		
Natural	4253/8353 (50.9)	1391/1637 (85.0)
Ovarian induction	430/8353 (5.1)	11/1637 (0.7)
Intrauterine insemination	914/8353 (10.9)	47/1637 (2.9)
*In‐vitro* fertilization	2716/8353 (32.5)	185/1637 (11.3)
Egg donation	40/8353 (0.5)	3/1637 (0.2)
Nulliparous	2620/5553 (47.2)	592/1249 (47.4)
Current smoker	586/8343 (7.0)	125/1550 (8.1)
Caucasian ethnicity	7835/8235 (95.1)	1523/1637 (93.0)

Data are given as *n*, median (interquartile range) or *n*/*N* (%).

* Data missing for 199 dichorionic (DC) and 39 monochorionic diamniotic (MCDA) twin pregnancies.

BMI, body mass index.

### Validation of dichorionic twin model

The DC twin cohort comprised 17 084 fetuses evaluated at a total of 95 346 eligible ultrasound scans. Among these scans, 68.4% (*n* = 65 209) were performed at scheduled visits and 44.5% (*n* = 42 453) were performed at scheduled visits in pregnancies carried to 37 + 0 weeks or later. The median number of scans per fetus from 20 + 0 weeks onwards was four (interquartile range (IQR), 3–5).

**Figure 3:**
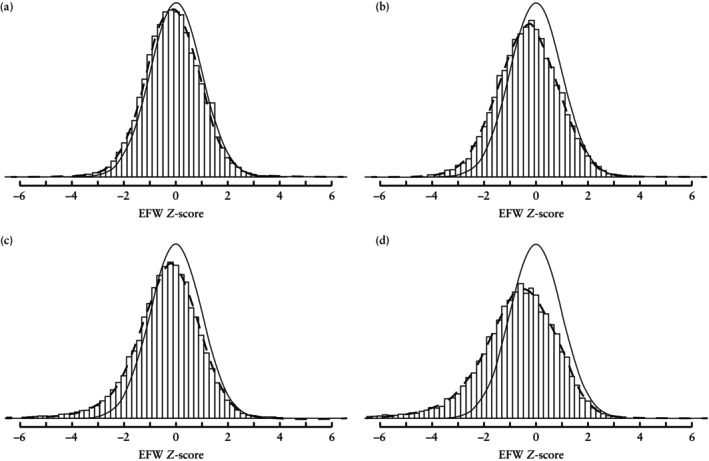
Distributional properties of Fetal Medicine Foundation models in Danish validation cohort of dichorionic twin pregnancies resulting in two liveborn children: (a) scheduled visits and delivery at or after 37 + 0 weeks; (b) non‐scheduled visits and delivery at or after 37 + 0 weeks; (c) scheduled visits and delivery before 37 + 0 weeks; and (d) non‐scheduled visits and delivery before 37 + 0 weeks. Smooth curve represents standard Gaussian distribution with a mean of 0 and SD of 1. Dashed line depicts estimated fetal weight (EFW) *Z*‐score distribution in validation cohort.

Figure 3 and Table 2 show the distributional properties of EFW *Z*‐scores derived from the model for DC twins. The model exhibited favorable alignment with the validation cohort for scheduled visits of DC twin pregnancies delivered at or after 37 + 0 weeks (mean ± SD EFW *Z*‐score, –0.14 ± 1.05). However, for non‐scheduled visits of pregnancies delivered at or after 37 + 0 weeks, a larger proportion of *Z*‐scores was observed in the lower range, indicating a left shift in the distribution (mean ± SD EFW *Z*‐score, –0.31 ± 1.17). Furthermore, pregnancies delivered before 37 + 0 weeks, both for scheduled visits (mean ± SD EFW *Z*‐score, –0.35 ± 1.31) and non‐scheduled visits (mean ± SD EFW *Z*‐score, –0.69 ± 1.50), exhibited even smaller weight estimates (*Z*‐scores), corresponding to a further left shift in the *Z*‐score distributions. These findings align with our definition of complicated DC pregnancy, namely one characterized by non‐scheduled visits and delivery before 37 + 0 weeks. Figure S1 provides a visual representation of the distributional properties of *Z*‐scores at each 4‐weekly scheduled visit and the overall distribution for scheduled visits in pregnancies delivered at 37 + 0 weeks or later.

**Table 2:** Estimated fetal weight *Z*‐scores in validation cohort, according to gestational age at delivery and type of visit

	DC twin pregnancy				MCDA twin pregnancy			
	Delivery < 37 + 0 weeks		Delivery ≥ 37 + 0 weeks		Delivery < 36 + 0 weeks		Delivery ≥ 36 + 0 weeks	
Visit	*n*	*Z*‐score	*n*	*Z*‐score	*n*	*Z*‐score	*n*	*Z*‐score
Scheduled	22 756	–0.35 ± 1.31	42 453	–0.14 ± 1.05	10 118	–0.25 ± 1.29	18 816	–0.09 ± 1.01
Non‐scheduled	11 513	–0.69 ± 1.50	18 624	–0.31 ± 1.17	1338	–0.63 ± 1.68	1360	–0.44 ± 1.22

*Z*‐scores are given as mean ± SD.

DC, dichorionic; MCDA, monochorionic diamniotic.

### Validation of monochorionic diamniotic twin model

The cohort of MCDA twins included 3350 fetuses assessed during 31 632 eligible ultrasound scans. Among these scans, 91.5% (*n* = 28 934) were performed at scheduled visits and 59.5% (*n* = 18 816) were performed at scheduled visits in pregnancies carried to 36 + 0 weeks or later. The median number of scans per fetus from 16 + 0 weeks onwards was 10 (IQR, 8–12).

**Figure 4:**
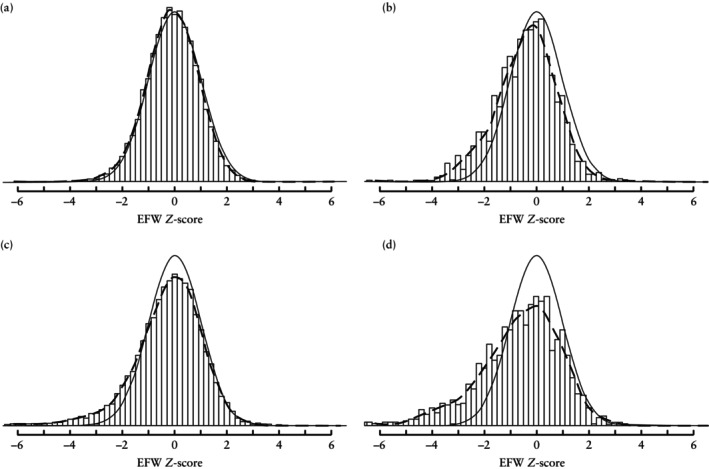
Distributional properties of Fetal Medicine Foundation models in Danish validation cohort of monochorionic diamniotic twin pregnancies resulting in two liveborn children: (a) scheduled visits and delivery at or after 36 + 0 weeks; (b) non‐scheduled visits and delivery at or after 36 + 0 weeks; (c) scheduled visits and delivery before 36 + 0 weeks; and (d) non‐scheduled visits and delivery before 36 + 0 weeks. Smooth curve represents standard Gaussian distribution with a mean of 0 and SD of 1. Dashed line depicts estimated fetal weight (EFW) *Z*‐score distribution in validation cohort.

Figure 4 and Table 2 display the distributional properties of EFW *Z*‐scores in the validation cohort using the model for MCDA twins. The model exhibited favorable alignment with the validation cohort for scheduled visits of MCDA twin pregnancies delivered at 36 + 0 weeks or later (mean ± SD EFW *Z*‐score, –0.09 ± 1.01). However, for non‐scheduled visits of pregnancies delivered at 36 + 0 weeks or later, a larger proportion of *Z*‐scores was observed in the lower range, indicating a left shift in the distribution (mean ± SD EFW *Z*‐score, –0.44 ± 1.22). For pregnancies delivered before 36 + 0 weeks, weight estimates (*Z*‐scores) for scheduled visits showed a less pronounced decrease (mean ± SD EFW *Z*‐score, –0.25 ± 1.29), while non‐scheduled visits of pregnancies delivered before 36 + 0 weeks displayed the highest proportion of small weight estimates (mean ± SD EFW *Z*‐score, –0.63 ± 1.68). These findings were consistent with our definition of complicated MCDA pregnancy, namely one characterized by non‐scheduled visits and delivery before 36 + 0 weeks. Figure S2 shows the distributional properties of *Z*‐scores at all scheduled visits and at each 2‐weekly scheduled visit in pregnancies delivered at 36 + 0 weeks or later.

## DISCUSSION

### Principal findings

This study demonstrates the validity of the FMF models for chorionicity‐specific fetal growth in twin pregnancies. For DC twins, the model showed favorable agreement with the validation cohort for scheduled visits in pregnancies delivered at 37 + 0 weeks or later. However, non‐scheduled visits and deliveries before 37 + 0 weeks exhibited a higher proportion of smaller weight estimates. Similar trends were observed for MCDA twins, with favorable agreement between the model and the validation cohort for scheduled visits in pregnancies delivered at 36 + 0 weeks or later. Non‐scheduled visits and deliveries before 36 + 0 weeks had a greater proportion of small weight estimates.

### Comparison with other studies

In the development study, Wright *et al*.^10^ established the FMF chorionicity‐specific models for fetal growth in twin pregnancies. These models were derived from an extensive cohort of twin pregnancies pooled from four institutions across three countries. The criteria for model development included uncomplicated pregnancies and scheduled visits, as per the definitions in the present study. Following the development of the models, Wright *et al*. published a study utilizing data from 1194 twin pregnancies and 29 035 singleton pregnancies in the EVENTS trial^28^ and investigated the use of singleton *vs* twin‐specific growth charts at 36 weeks^29^. The findings indicated that twins classified as SGA using singleton charts exhibited higher neonatal morbidity. This demonstrates the superiority of singleton charts in classifying and detecting growth‐related risks, supporting their use for consistent fetal‐growth classification in both twins and singletons. Other investigators recommend enhanced precision in detecting adverse outcome through the application of twin‐specific growth charts^30^, ^31^. These studies are limited by their relatively small sample sizes, resulting in a lack of statistical power to address conclusively the relatively infrequent outcomes observed. Moreover, they fail to address the potential oversight in identifying adverse outcomes among pregnancies categorized as AGA based on the twin‐specific chart but as SGA based on the singleton chart.

Previous research on fetal growth in twin pregnancies has demonstrated a lack of consistency, utilizing various methods and differing definitions for categorizing pregnancies as complicated or uncomplicated^13^, ^32^, ^33^, ^34^, ^35^, ^36^, ^37^, ^38^, ^39^, ^40^, ^41^, ^42^. This includes varying approaches to excluding complicated pregnancies, thus adding another layer of uncertainty and complicating direct comparisons between studies. Most importantly, to our knowledge, none of these studies has been validated externally, limiting their generalizability^13^, ^32^, ^33^, ^34^, ^35^, ^36^, ^37^, ^38^, ^39^, ^40^, ^41^, ^42^. There has been no prospective study to date that examined the consequences of implementing growth charts specifically designed for twins.

### Clinical implications

This external validation of the FMF models for chorionicity‐specific fetal growth in twins has several clinical implications. First, our study shows a strong level of agreement between the FMF models and the largest detailed national dataset of DC and MCDA twin pregnancies documented to date. This evidence supports the use of the FMF models as a novel, standardized approach for evaluating fetal growth in twin pregnancies. This approach involves combining and interpreting chorionicity‐specific fetal growth in twins relative to fetal growth in singletons. Adopting this method could potentially enhance the accuracy and scientific integrity of fetal‐growth evaluation by avoiding the normalization of smallness in twins while still incorporating specific details of twin growth. Second, using the singleton percentile scale, the validated models offer an improved standardized approach for evaluating fetal growth in twins relative to singletons. This technique facilitates improved comparisons and more accurate identification of potential deviations from expected growth patterns, especially at earlier gestations. By doing so, the approach aims to reduce unnecessary visits and interventions in fetal monitoring while ensuring that cases at risk are not overlooked. However, these associations have not yet been proven and should be a focus for future research to confirm this method's effectiveness and safety. Third, the validated models improve our understanding of fetal growth in twin pregnancies by potentially aiding in the identification of abnormal growth trajectories. These trajectories do not correspond to either a twin‐specific or a singleton reference but contain features of both. Identifying such trajectories could be crucial in signaling underlying complications or increased risk of adverse outcome.

### Strengths and limitations

This study has several strengths. First, the external validation was performed in a national Danish cohort of, to our knowledge, the largest highly detailed dataset of DC and MCDA twin pregnancies. The study benefits from a reduced risk of selection bias, owing to the detailed and comprehensive data provided by the Danish registries. The multicenter development cohort and national validation cohort enhance the generalizability of our findings. Additionally, prenatal assessments in the validation cohort were performed in a setting with a very high participation rate covered by the Danish tax‐funded public healthcare system for all pregnant women, ensuring equal access to care. The models used in this study are based on the MCMC method, effectively incorporating the relationships between interpregnancy, interfetal and intervisit factors. The reference cohort was well defined, comprising uncomplicated pregnancies resulting in two liveborn children, seen for scheduled visits and delivered at or after the internationally recommended gestational age. The study also successfully demonstrated differences in pregnancies delivered earlier than the reference cohort and those seen for non‐scheduled visits.

This study is subject to certain limitations. First, it utilizes retrospective development and validation cohorts, which could introduce inherent biases. Second, the models are exclusively based on EFW calculated using the Hadlock‐3 formula. Apart from their use in the Hadlock‐3 formula, detailed individual biometric measurements (head circumference, abdominal circumference and femur length) for each twin are not utilized in the models, nor do the models include Doppler measurements or amniotic fluid assessment. Third, the validation cohort consisted predominantly of Caucasian women with normal body mass index, which may limit the applicability of the findings to diverse populations. Fourth, the models used in this study, similar to the FMF models for singletons, do not account for fetal sex, a factor known to influence growth patterns. This exclusion may limit the precision of fetal‐growth assessment, as male and female fetuses typically follow different growth trajectories. Fifth, chorionicity is crucial for accurate monitoring protocols but was not routinely confirmed by postpartum examination of the placentae in our cohort. In instances of unresolved uncertainty regarding chorionicity, pregnancies were managed as monochorionic, adhering to the more intensive monitoring protocol applicable to these pregnancies. Sixth, categorizing non‐scheduled visits as indicative of complicated pregnancy may be an oversimplification, as these visits could result from suspected issues that warranted additional monitoring rather than confirmed complications. Last, the inclusion of different healthcare systems in the development and validation cohorts might have introduced variability in care and outcomes, potentially impacting on the generalizability of the study findings in various healthcare settings.

### Conclusions

The results of this external validation study, based on a large Danish nationwide cohort of twin pregnancies, reinforce the validity of the chorionicity‐specific FMF models for describing fetal growth in twin pregnancies. By juxtaposing singleton and chorionicity‐specific twin growth distributions, the models provide a more nuanced and detailed evaluation of fetal growth in twins. This study provides robust evidence validating the FMF models as reliable references for understanding fetal growth in twin pregnancies.

It is imperative to establish associations between the FMF models, pregnancy outcomes and long‐term outcomes for the children. Furthermore, future studies should explore the integration of Doppler measurements, biomarkers and other relevant factors of importance to fetal health and development. Through these efforts, we hope to develop more precise, evidence‐based management strategies, providing the best possible health trajectory for these high‐risk pregnancies and enhancing the standard of obstetric care.

## Supporting information


**Figure S1** Distributional properties of Fetal Medicine Foundation models in Danish validation cohort of dichorionic twin pregnancies delivering two liveborn children at or after 37 + 0 weeks' gestation, at each of the 4‐weekly scheduled visits. Smooth curve represents standard Gaussian distribution with mean of 0 and SD of 1. Dashed red line depicts *Z*‐score distribution in validation cohort.

**Figure S2** Distributional properties of Fetal Medicine Foundation models in Danish validation cohort of monochorionic diamniotic twin pregnancies delivering two liveborn children at or after 36 + 0 weeks' gestation, at each of the 2‐weekly scheduled visits. Smooth curve represents standard Gaussian distribution with mean of 0 and SD of 1. Dashed red line depicts *Z*‐score distribution in validation cohort.

## Data Availability

The pregnancy outcome dataset analyzed in this study is available upon application to the Danish Fetal Medicine Database (FØTO). Access to this database is overseen by the Danish Clinical Quality Program‐ National Clinical Registries, which controls the use of the data to ensure patient confidentiality and appropriate use of the data. Information on the data application process and criteria can be found at https://www.rkkp.dk/forskning/ (in Danish).
