# Supplementary figures and images for: Validation of Fetal Medicine Foundation charts for fetal growth in twins: nationwide Danish cohort study
Source: Ultrasound Obstet Gynecol. 2024 Oct 27;64(6):730–8. doi: 10.1002/uog.29125 (PMC11609905; doi:10.1002/uog.29125)

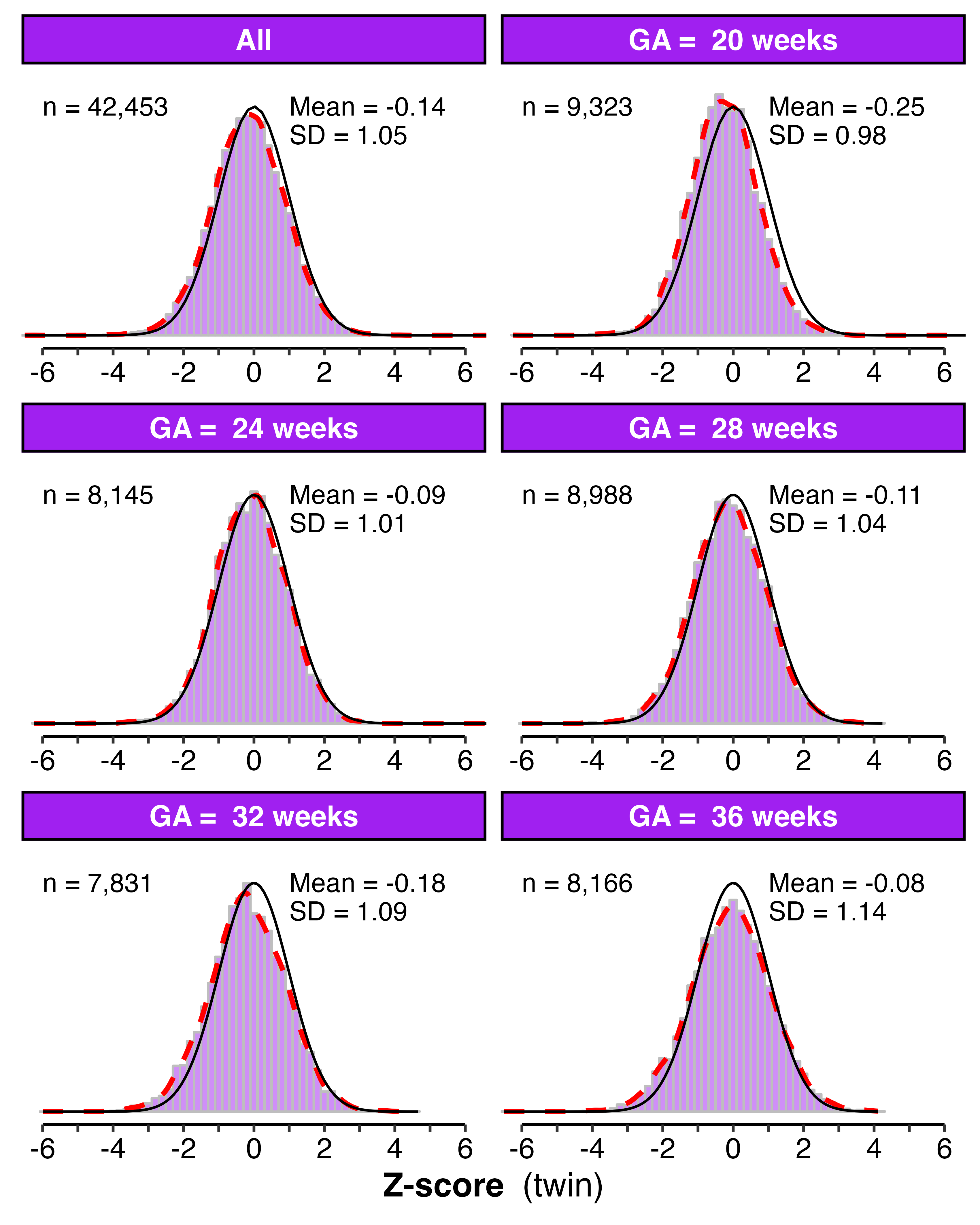

Supplement: Supplementary file 1 — Figure S1 Distributional properties of Fetal Medicine Foundation models in Danish validation cohort of dichorionic twin pregnancies delivering two liveborn children at or after 37 + 0 weeks' gestation, at each of the 4‐weekly scheduled visits. Smooth curve represents standard Gaussian distribution with mean of 0 and SD of 1. Dashed red line depicts Z‐score distribution in validation cohort. [file UOG-64-730-s002.png]

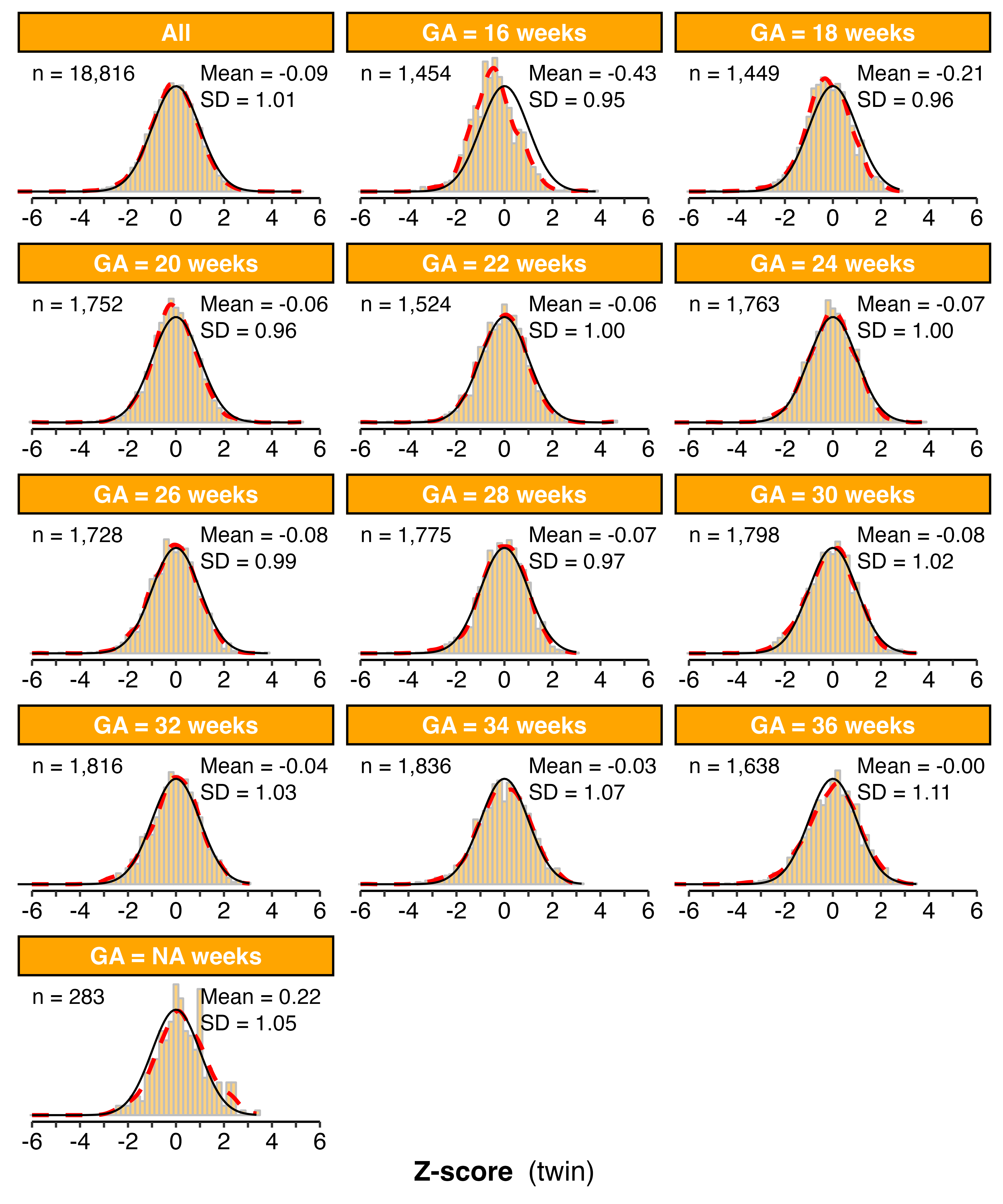

Supplement: Supplementary file 2 — Figure S2 Distributional properties of Fetal Medicine Foundation models in Danish validation cohort of monochorionic diamniotic twin pregnancies delivering two liveborn children at or after 36 + 0 weeks' gestation, at each of the 2‐weekly scheduled visits. Smooth curve represents standard Gaussian distribution with mean of 0 and SD of 1. Dashed red line depicts Z‐score distribution in validation cohort. [file UOG-64-730-s001.png]
